# Gastrointestinal Cancer Stage at Diagnosis Before and During the COVID-19 Pandemic in Japan

**DOI:** 10.1001/jamanetworkopen.2021.26334

**Published:** 2021-09-21

**Authors:** Kento Kuzuu, Noboru Misawa, Keiichi Ashikari, Takaomi Kessoku, Shingo Kato, Kunihiro Hosono, Masato Yoneda, Takashi Nonaka, Shozo Matsushima, Tatsuji Komatsu, Atsushi Nakajima, Takuma Higurashi

**Affiliations:** 1 Department of Gastroenterology and Hepatology, Yokohama City University School of Medicine, Kanazawa-ku, Yokohama, Japan; 2 National Hospital Organization Yokohama Medical Center, Totuka-ku, Yokohama, Japan

## Abstract

**Importance:**

The COVID-19 pandemic has delayed medical consultations, possibly leading to the diagnosis of gastrointestinal cancer at advanced stages.

**Objective:**

To evaluate stage at diagnosis among patients with gastrointestinal cancer in Japan before and during the COVID-19 pandemic.

**Design, Setting, and Participants:**

This retrospective cohort study included patients in a hospital-based cancer registry who were diagnosed with gastrointestinal cancer (ie, esophageal, gastric, colorectal, pancreatic, liver, and biliary tract cancers) between January 2016 and December 2020 at 2 tertiary Japanese hospitals.

**Exposures:**

The pre–COVID-19 period was defined as January 2017 to February 2020, and the COVID-19 period was defined as March 2020 to December 2020.

**Main Outcome and Measure:**

Monthly numbers of patients with newly diagnosed cancer were aggregated, classified by stage, and compared.

**Results:**

The study evaluated 5167 patients, including 4218 patients (2825 [67.0%] men; mean [SD] age, 71.3 [10.9] years) in the pre–COVID-19 period and 949 patients (607 [64.0%] men; mean [SD] age, 71.8 [10.7] years) in the COVID-19 period. Comparing the pre–COVID-19 period with the COVID-19 period, significant decreases were observed in the mean (SD) number of patients with newly diagnosed gastric cancer (30.63 [6.62] patients/month vs 22.40 [5.85] patients/month; –26.87% change; *P* < .001) and colorectal cancer (41.61 [6.81] patients/month vs 36.00 [6.72] patients/month; –13.47% change; *P* = .03). Significant decreases were also observed in the mean (SD) number of cases of stage I gastric cancer (21.55 [5.66] cases/month vs 13.90 [5.99] cases/month; –35.51% change; *P* < .001), stage 0 colorectal cancer (10.58 [3.36] cases/month vs 7.10 [4.10] cases/month; –32.89% change; *P* = .008), and stage I colorectal cancer (10.16 [3.14] cases/month vs 6.70 [2.91] cases/month; –34.04% change; *P* = .003). No significant increases were observed for esophageal, gastric, pancreatic, liver, or biliary tract cancers. A significant decrease was observed in the mean (SD) number of cases per month of stage II colorectal cancer (7.42 [3.06] cases/month vs 4.80 [1.75] cases/month; –35.32% change; *P* = .01); a significant increase was observed for the mean (SD) number of cases per month of stage III colorectal cancer (7.18 [2.85] cases/month vs 12.10 [2.42] cases/month; 68.42% change; *P* < .001).

**Conclusions and Relevance:**

In this cohort study of patients in a hospital-based cancer registry form Japan, significantly fewer patients were diagnosed with stage I gastric and colorectal cancers during the COVID-19 pandemic. Thus, the number of screening-detected cancers might have decreased, and colorectal cancer may have been diagnosed at more advanced stages.

## Key Points

**Question:**

Is the COVID-19 pandemic associated with the stage at which gastrointestinal cancer is diagnosed in Japan?

**Findings:**

In this cohort study of 5167 patients, significant decreases were observed for the diagnosis of stage I gastric cancer and stage 0 to II colorectal cancer, whereas a significant increase was observed for the diagnosis of stage III colorectal cancer.

**Meaning:**

These findings suggest that during the COVID-19 pandemic, there may have been fewer cases of screening-detected gastrointestinal cancer, and colorectal cancer may have been diagnosed at more advanced stages.

## Teaser

This cohort study evaluates stage at diagnosis among patients with gastrointestinal cancer in Japan before and during the COVID-19 pandemic.

## Introduction

COVID-19 is caused by SARS-CoV-2. The first case was identified in Wuhan, China, during December 2019, and the disease has subsequently spread worldwide, leading to an ongoing pandemic. The symptoms of COVID-19 are variable but often include fever, cough, difficulty breathing, and lost senses of smell and taste. At least one-third of people infected with SARS-CoV-2 remain asymptomatic but are able to spread the virus,^1,2^ which primarily occurs via close personal contact.^3,4^ Therefore, many governments have implemented lockdowns or declared states of emergency to prevent the spread of SARS-CoV-2.^5^

The first Japanese case of COVID-19 was confirmed on January 16, 2020, which was followed by outbreaks on the Diamond Princess cruise ship, restaurants, and nursing homes in February 2020. The number of infected individuals in Japan continued to increase, which prompted school closures and the cancellation of large-scale events in March 2020, along with calls for self-quarantine and stay-at-home measures. On April 7, 2020, the Japanese government proclaimed a state of emergency, which was subsequently lifted on May 25, 2020. However, the total number of confirmed cases in Japan exceeded 400 000 on February 6, 2021, and social distancing, self-quarantine, and stay-at-home measures are still required.

While measures are needed to control the number of new COVID-19 cases,^6^ these measures can negatively affect the diagnosis of other major medical conditions. For example, previous studies have shown that the COVID-19 pandemic was associated with decreased diagnostic rates for acute heart failure,^7^ stroke,^8^ and pulmonary embolism,^9^ which may be related to decreased consultations for patients with relatively minor illnesses and symptoms. Other studies have also shown that the incidence of cancer decreased during the pandemic in several countries.^10,11,12,13,14,15,16,17^ Furthermore, a cross-sectional study conducted in the United States revealed that weekly diagnoses during the pandemic decreased by 46.4% for 6 cancers (breast, colorectal, lung, pancreatic, gastric, and esophageal cancers), with significant decreases for all cancer types, ranging from a decrease of 24.7% for pancreatic cancer to 51.8% for breast cancer.^10^ Moreover, a UK study regarding endoscopic activity and cancer detection during the COVID-19 pandemic revealed a decrease of 12% (vs pre–COVID-19 levels) in endoscopic activity and a decrease of 58% in weekly cancer diagnoses, with specific reductions of 19% for pancreatobiliary cancers, 37% for esophageal cancers, 52% for gastric cancers, and 72% for colorectal cancers.^11^

Unfortunately, measures that aim to control the COVID-19 pandemic do not stop cancer progression and could lead to diagnosis at more advanced stages and thus poorer clinical outcomes. One study estimated that COVID-19–related measures may lead to an extra 33 890 cancer-related deaths in the United States.^18^ Furthermore, by the fifth year after diagnosis, the number of colorectal cancer–related deaths may increase by 15.3% to 16.6% and the number of esophageal cancer–related deaths may increase by 4.8% to 5.3%.^19^ Therefore, this study aimed to evaluate whether gastrointestinal cancers were diagnosed at more advanced stages during the COVID-19 pandemic in Japan. To our knowledge, this is the first study to evaluate whether the COVID-19 pandemic has affected the stage of gastrointestinal cancer at diagnosis in Japan.

## Methods

### Study Design and Setting

This retrospective study evaluated patients who were treated at the Yokohama City University Hospital and the National Hospital Organization Yokohama Medical Center between January 2017 and December 2020. Despite caring for patients with severe COVID-19, neither hospital had a cluster outbreak^20^; therefore, these 2 hospitals did not restrict consultation, examination, surgery, or chemotherapy. However, the Japan Gastroenterological Endoscopy Society recommended that gastroscopy and colonoscopy be postponed in asymptomatic patients until after the state of emergency (April and May 2020) due to the high risk of infection these procedures carry, which these hospitals complied with. This report follows the Strengthening the Reporting of Observational Studies in Epidemiology (STROBE) guidelines. The retrospective study protocol was approved by the ethics committees of Yokohama City University Hospital (November 17, 2020) and National Hospital Organization Yokohama Medical Center (October 13, 2020). Patients could opt out of the study via the hospitals’ websites.

### Patients and Cancer Registry

Patients were evaluated if they had a new diagnosis of gastrointestinal cancer between January 2017 and December 2020. Patients with cancer were identified from hospital-based cancer registries that are part of the National Cancer Registry.^21^ The cancer registries are registered by registrants who are certified by the National Cancer Center Japan. Patients with suspected cancer were identified and aggregated into a single list based on disease name, pathological findings, surgical history, chemotherapy history, palliative care history, radiation therapy history, and history of referral to cancer centers. The patients’ records were then searched to collect detailed information. Gastrointestinal cancers were defined according to the *International Statistical Classification of Diseases and Related Health Problems, Tenth Revision *(*ICD-10*) codes: esophageal cancer, C15; gastric cancer, C16; colorectal cancer, C18 to C20; hepatocellular carcinoma, C22; biliary tract cancer, C23 to C24; and pancreatic cancer, C25. Gastrointestinal cancers did not include neuroendocrine tumors and lymphomas. Furthermore, intrahepatic cholangiocarcinoma was excluded because it is clinically and pathologically distinct from hepatocellular carcinoma (*ICD-10 *code, C22). Patients were considered eligible if they were diagnosed or started their first treatment (including palliative care) at the study hospitals and were considered ineligible if they were diagnosed and started their first treatment at other hospitals.

### Data Extraction

Data were extracted regarding patient sex, age, date of diagnosis, process of detection, clinical stage (based on the Union for International Cancer Control staging system, eighth edition), and first treatment. Patients whose records did not clearly identify the cancer stage and/or first treatment were reregistered based on information from their medical records. The date of diagnosis was defined as the date of the first clinical examination performed to diagnose cancer (not the date of the pathological diagnosis).

### Outcomes

The period before COVID-19 was defined as January 2017 to February 2020, while the period during the COVID-19 pandemic in Japan was defined as March 2020 to December 2020. We aggregated the numbers of patients with newly diagnosed cancer for monthly periods, classified them according to stage, and then compared the monthly numbers. The patients’ diseases were also classified as localized stage (the cancer was confined to the original organ), regional stage (the cancer had spread to the regional lymph nodes and/or immediately adjacent tissues), or distant stage (the cancer had metastasized to distant organs).^22^ The detection process was categorized as medical checkup (conducted by a company, school, or municipality or at the patient’s own expense), screening (for patients with no symptoms being treated for other diseases), and symptomatic cases.

### Statistical Analysis

Patient characteristics were compared using the χ^2^ test, *t* test, or Mann-Whitney *U* test, as appropriate. Monthly case counts were compared using the *t* test. Differences were considered statistically significant at *P* <.05, and all tests were 2-tailed. All statistical analyses were performed using BellCurve 3.20 (Social Survey Research Information) for Excel 2016 (Microsoft Corp).

## Results

### Patient Characteristics

The study evaluated 5167 patients, including 4218 patients from before the COVID-19 pandemic and 949 patients diagnosed during the COVID-19 pandemic in Japan (Table 1 and Table 2). There were no significant differences between the 2 periods in terms of the proportions of male patients (2825 patients [67.0%] vs 607 patients [64.0%]; *P* = .08) or mean (SD) age (71.3 [10.9] years vs 71.8 [10.7] years; *P* = .19). There were also no significant differences in mean ages and sex ratios according to cancer type.

**Table 1.** Patient Characteristics

Characteristic	Patients, No. (%)		*P* value^b^
	Before COVID-19^a^	During COVID-19^a^	
All gastrointestinal cancers			
Total No.	4218	949	NA
Age, mean (SD), y	71.3 (10.9)	71.8 (10.7)	.19
Men	2825 (67.0)	607 (64.0)	.08
Women	1393 (33.0)	342 (36.0)	
Colorectal cancer			
Total No.	1581	360	NA
Age, mean (SD), y	70.4 (11.6)	70.8 (11.6)	.58
Men	978 (61.9)	208 (57.8)	.15
Women	603 (38.1)	152 (42.2)	
Medical checkup cases^c^	207 (13.1)	41 (11.4)	.60
Screening cases^c^	526 (33.3)	101 (28.1)	.17
Symptomatic cases^c^	796 (50.3)	205 (56.9)	.21
Gastric cancer			
Total No.	1164	224	NA
Age, mean (SD), y	72.5 (10.0)	73.5 (9.3)	.17
Men	836 (71.8)	160 (71.4)	.91
Women	328 (28.2)	64 (28.6)	
Medical checkup cases^c^	212 (18.2)	37 (16.5)	.61
Screening cases^c^	493 (42.4)	80 (35.7)	.23
Symptomatic cases^c^	406 (34.9)	102 (45.5)	.04
Pancreatic cancer			
Total No.	532	141	NA
Age, mean (SD), y	69.4 (11.5)	71.0 (10.8)	.13
Men	311(58.5)	73(51.8)	.15
Women	221 (41.5)	68 (48.2)	
Medical checkup cases^c^	38 (7.1)	8 (5.7)	.56
Screening cases^c^	154 (28.9)	37 (26.2)	.63
Symptomatic cases^c^	320 (60.2)	90 (63.8)	.70
Esophageal cancer			
Total No.	335	87	NA
Age, mean (SD), y	71.6 (9.1)	71.8 (10.0)	.90
Men	275 (82.1)	68 (78.2)	.40
Women	60 (17.9)	19 (21.8)	
Medical checkup cases^c^	37 (11.0)	11 (12.6)	.92
Screening cases^c^	129 (38.5)	30 (34.5)	.64
Symptomatic cases^c^	146 (43.6)	40 (46.0)	.71
Hepatocellular carcinoma			
Total No.	338	75	NA
Age, mean (SD), y	73.5 (10.8)	72.5 (10.4)	.48
Men	244 (72.2)	57 (76.0)	.50
Women	94 (27.8)	18 (24.0)	
Medical checkup cases^c^	21 (6.2)	8 (10.7)	.21
Screening cases^c^	249 (73.7)	46 (61.3)	.37
Symptomatic cases^c^	59 (17.5)	17 (22.7)	.39
Biliary tract cancer			
Total No.	268	62	NA
Age, mean (SD), y	72.2 (10.2)	72.8 (10.6)	.66
Men	181 (67.5)	41 (66.1)	.83
Women	87 (32.5)	21 (33.9)	
Medical checkup cases^c^	17 (6.3)	4 (6.5)	.98
Screening cases^c^	73 (27.2)	13 (21.0)	.43
Symptomatic cases^c^	168 (62.7)	44 (71.0)	.57

Abbreviation: NA, not applicable.

^a^ The period before COVID-19 was defined as January 2017 to February 2020, and the period during COVID-19 was defined as March to December 2020.

^b^ *P* values were calculated using the *t* test or χ^2^ test.

^c^ The process of detection was categorized into medical checkup (conducted by the company, school, or municipality or at the patient’s own expense), screening (for patients with no symptoms being treated for other diseases), and symptomatic cases.

**Table 2.** Comparison of the Numbers of Patients With Gastroenterology Visits, Gastroscopies, and Endoscopies Before and During the COVID-19 Pandemic

Appointment	Cumulative, No.		Per month, mean (SD)		Change, %^a^	*P* value^b^
	Before	During	Before	During		
First visit	14 990	3321	394 (33.8)	332 (88.3)	−15.74	.02
Return visit	169 418	41 282	4458 (279)	4128 (564)	−7.40	.14
Gastroscopy	27 974	6927	736 (13.7)	693 (39.4)	−5.90	.29
Colonoscopy	16 755	3989	441 (45.3)	399 (82.9)	−9.53	.21

^a^ Mean percentage change per month.

^b^ *P* values were calculated using the Mann-Whitney *U* test.

The detection processes were compared, and the proportion of all gastrointestinal cancer cases detected via symptoms increased, although this increase was only significant for gastric cancer cases (pre–COVID-19: 406 patients [34.9%]; during COVID-19: 102 patients [45.5%]; *P* = .04). There were no changes in the proportion of cases detected by medical checkup, but the proportion of cases detected by screening decreased despite not being statistically significant.

A total of 18 311 initial gastroenterology visits (pre–COVID-19: 14 990 visits; during COVID-19: 3321 visits) were identified, and the monthly number of initial visits decreased significantly from a mean (SD) of 394 (33.8) visits/month to 332 (88.3) visits/month (a –15.74% change; *P* = .02). The numbers of gastroscopies, colonoscopies, and patients who returned to undergo gastroenterology examinations decreased, although these decreases were not significant.

### Colorectal Cancer

There were 1941 cases of colorectal cancer (pre–COVID-19: 1581; during COVID-19: 360) (Table 3), and the monthly mean (SD) number of patients decreased significantly from 41.61 (6.81) patients/month to 36.00 (6.72) patients/month (a –13.47% change; *P* = .03). Significant decreases were observed in the mean (SD) number of diagnosed cases of stage 0 colorectal cancer (10.58 [3.36] cases/month vs 7.10 [4.10] cases/month; *P* = .008), stage I colorectal cancer (10.16 [3.14] cases/month vs 6.70 [2.91] cases/month; *P* = .003), and stage II colorectal cancer (7.42 [3.06] cases/month vs 4.80 [1.75] cases/month; *P* = .01). However, significant increases were observed in the mean (SD) number of diagnosed cases of stage III colorectal cancer (7.18 [2.85] cases/month vs 12.10 [2.42] cases/month; *P* < .001). Similarly, the mean (SD) number of cases diagnosed at the localized stage decreased (27.18 [5.28] cases/month vs 17.9 [6.42] cases/month; *P* < .001), whereas the mean (SD) number of cases diagnosed at the regional stage increased (8.16 [2.97] cases/month vs 12.8 [2.57] cases/month; *P* < .001).

**Table 3.** Comparison of the Numbers of Colorectal and Gastric Cancers Before and During the COVID-19 Pandemic

Cancer stage	Cumulative, No.		Per month, mean (SD)		Change, %	*P* value^a^
	Before	During	Before	During		
**Colorectal cancer**						
Total	1581	360	41.61 (6.81)	36.00 (6.72)	−13.47	.03
Stage 0	402	71	10.58 (3.36)	7.10 (4.10)	−32.89	.008
Stage I	386	67	10.16 (3.14)	6.70 (2.91)	−34.04	.003
Stage II	282	48	7.42 (3.06)	4.80 (1.75)	−35.32	.01
Stage III	273	121	7.18 (2.85)	12.10 (2.42)	68.42	<.001
Stage IV	238	53	6.26 (3.13)	5.30 (2.83)	−15.38	.38
Localized^b^	1033	179	27.18 (5.28)	17.9 (6.42)	−34.15	<.001
Regional^b^	310	128	8.16 (2.97)	12.8 (2.57)	56.90	<.001
Distant^b^	238	53	6.26 (3.13)	5.30 (2.83)	−15.38	.38
**Gastric cancer**						
Total	1164	224	30.63 (6.62)	22.40 (5.85)	−26.87	<.001
Stage I	819	139	21.55 (5.66)	13.90 (5.99)	−35.51	<.001
Stage II	103	22	2.71 (1.59)	2.20 (1.40)	−18.82	.36
Stage III	75	14	1.97 (1.37)	1.40 (1.08)	−29.07	.23
Stage IV	167	49	4.40 (0.33)	4.90 (3.52)	11.36	.67
Localized^c^	871	157	22.92 (5.95)	15.70 (6.45)	−31.50	.002
Regional^c^	143	24	3.76 (0.31)	2.40 (1.26)	−36.22	.037
Distant^c^	150	43	3.95 (1.99)	4.30 (2.95)	8.93	.66

^a^ *P* values were calculated using the *t* test.

^b^ Colorectal cancer was categorized as localized stage (stage 0, I, IIA, or IIB), regional stage (stage IIC, IIIA, IIIB, or IIIC), or distant stage (stage IVA, IVB, IVC).

^c^ Gastric cancer was categorized as localized stage (stage I or IIB), regional stage (stage IIA, III, or IVA), or distant stage (stage IVB).

### Gastric Cancer

There were 1388 cases of gastric cancer (pre–COVID-19: 1164; during COVID-19: 224) (Table 3), and the mean (SD) monthly number of patients diagnosed decreased significantly, from 30.63 (6.62) patients/month to 22.40 (5.85) patients/month (a –26.87% change; *P* < .001). A significant decrease was observed in the mean (SD) number of cases of stage I gastric cancer (21.55 [5.66] cases/month vs 13.90 [5.99] cases/month; *P* < .001). No significant changes were observed for stage II to stage IV gastric cancer, although an 11.36% increase (mean [SD] cases, pre–COVID-19: 4.40 [0.33] cases/month; during COVID-19: 4.90 [3.52] cases/month) was observed in the number of stage IV gastric cancer cases. Significant decreases were also observed in the mean (SD) number of cases at the localized stage (22.92 [5.95] cases/month vs 15.70 [6.45] cases/month, *P* = .002) and regional stage (3.76 [0.31] cases/month vs 2.40 [1.26] cases/month; *P* = .04).

### Pancreatic Cancer

There were 673 cases of pancreatic cancer (pre–COVID-19: 532; during COVID-19: 141) (Table 4); no significant change was noted in the mean (SD) monthly number of patients (14.00 [3.37] patients/month vs 14.10 [3.32] patients/month; *P* = .93). Although there were no significant differences, the number of patients with stage I or II disease decreased, whereas the number of patients with stage III or IV disease increased.

**Table 4.** Comparison of the Numbers of Pancreatic and Esophageal Cancers Before and During the COVID-19 Pandemic

Cancer stage	Cumulative, No.		Per month, mean (SD)		Change, %	*P* value
	Before	During	Before	During		
**Pancreatic cancer**						
Total	532	141	14.00 (3.37)	14.10 (3.32)	0.71	.93
Stage 0	19	8	0.50 (0.60)	0.80 (0.79)	60.00	.20
Stage I	127	25	3.34 (2.45)	2.50 (1.58)	−25.20	.31
Stage II	93	24	2.45 (1.54)	2.40 (1.71)	−1.94	.93
Stage III	79	23	2.08 (1.53)	2.30 (1.64)	10.63	.70
Stage IV	214	61	5.63 (2.31)	6.10 (2.51)	8.32	.58
Localized^a^	206	42	5.42 (2.68)	4.20 (1.81)	−22.52	.18
Regional^a^	112	38	2.95 (2.19)	3.80 (1.87)	28.93	.27
Distant^a^	214	61	5.63 (2.31)	6.10 (2.51)	8.32	.58
**Esophageal cancer**						
Total	335	87	8.82 (2.75)	8.70 (2.71)	−1.31	.91
Stage 0	49	9	1.29 (0.96)	0.90 (0.74)	−30.20	.24
Stage I	120	31	3.16 (1.52)	3.10 (1.45)	−1.83	.91
Stage II	37	8	0.97 (1.03)	0.80 (0.79)	−17.84	.62
Stage III	50	15	1.32 (1.23)	1.50 (1.27)	14.00	.68
Stage IV	79	24	2.08 (0.26)	2.40 (0.45)	15.44	.57
Localized^b^	206	48	5.42 (2.01)	4.80 (1.48)	−11.46	.37
Regional^b^	78	27	2.05 (0.27)	2.70 (0.54)	31.54	.28
Distant^b^	51	12	1.34 (0.23)	1.20 (0.42)	−10.59	.77

^a^ Pancreatic cancer was categorized as localized stage (stage 0, IA, IB, or IIA), regional stage (stage IIB or III), or distant stage (stage IV).

^b^ Esophageal cancer was categorized as localized stage (stage 0, I, or II), regional stage (stage III or IVA), or distant stage (stage IVB).

### Esophageal Cancer

There were 422 cases of esophageal cancer (pre–COVID-19: 335; during COVID-19: 87) (Table 4), and no significant change was noted in the mean (SD) monthly number of patients (8.82 [2.75] patients/month vs 8.70 [2.71] patients/month, *P* = .91). Although there were no significant differences, the number of patients with stage 0 to II disease decreased, whereas the number of patients with stage III or IV disease increased.

### Hepatocellular Carcinoma

There were 413 cases of hepatocellular carcinoma (pre–COVID-19: 338; during COVID-19: 75) (Table 5); a nonsignificant decrease was noted in the mean (SD) monthly number of patients (8.89 [3.17] patients/month vs 7.50 [2.27] patients/month; –15.68% change; *P* = .20). No significant difference was observed according to stage, although a –33.22% change was observed in the mean (SD) number of stage I hepatocellular carcinoma cases (4.87 [2.28] cases/month vs 3.30 [2.06] cases/month, *P* = .06). As with most other cancer types, although there were no significant differences, the number of patients with stage I or II disease decreased, whereas the number of patients with Stage III or IV disease increased.

**Table 5.** Numbers of Hepatocellular Carcinomas and Biliary Tract Cancers Before and During the COVID-19 Pandemic

Cancer stage	Cumulative, No.		Per month, mean (SD)		Change, %	*P* value
	Before	During	Before	During		
**Hepatocellular carcinoma**						
Total	338	75	8.89 (3.17)	7.50 (2.27)	−15.68	.20
Stage I	185	33	4.87 (2.28)	3.30 (2.06)	−32.22	.06
Stage II	70	17	1.84 (1.72)	1.70 (1.49)	−7.71	.81
Stage III	51	16	1.34 (1.12)	1.60 (1.71)	19.22	.66
Stage IV	32	9	0.84 (0.92)	0.90 (1.10)	6.88	.87
Localized^a^	286	59	7.53 (2.76)	5.90 (2.08)	−21.61	.09
Regional^a^	30	12	0.79 (0.94)	1.20 (0.59)	52.00	.33
Distant^a^	22	4	0.58 (0.76)	0.40 (0.70)	−30.91	.50
**Biliary tract cancer**						
Total	268	62	7.05 (3.06)	6.20 (2.25)	−12.09	.42
Stage 0	13	3	0.34 (0.52)	0.30 (0.68)	−12.31	.85
Stage I	49	11	1.29 (1.41)	1.10 (0.99)	−14.69	.69
Stage II	70	23	1.84 (1.48)	2.30 (1.64)	24.86	.40
Stage III	59	12	1.55 (1.37)	1.20 (1.03)	−22.71	.45
Stage IV	77	13	2.03 (1.64)	1.30 (1.16)	−35.84	.20
Localized^b^	103	25	2.71 (1.89)	2.50 (1.90)	−7.77	.76
Regional^b^	92	25	2.42 (1.46)	2.50 (0.85)	3.26	.87
Distant^b^	73	12	1.92 (1.58)	1.20 (1.23)	−37.53	.19

^a^ Hepatocellular carcinoma was categorized as localized stage (stage IA, IB, II, or IIIA), regional stage (stage IIIB or IVA), or distant stage (stage IVB).

^b^ Gallbladder cancer was categorized as localized stage (stage 0, IA, IB), regional stage (stage IIA, IIB, IIIA, IIIB, or IVA), or distant stage (stage IVB). Hilar cholangiocarcinoma was categorized as localized stage (stage 0 or I), regional stage (stage II, IIIA, IIIB, IIIC, or IVA), or distant stage (stage IVB). Distal cholangiocarcinoma was categorized as localized stage (stage 0, I, or IIA), regional stage (stage IIB, IIIA, or IIIB), or distant stage (stage IV).

### Biliary Tract Cancer

There were 330 cases of biliary tract cancer (pre–COVID-19: 268; during COVID-19: 62) (Table 5), and a nonsignificant decrease was noted in the mean (SD) monthly number of patients (7.05 [3.06] patients/month vs 6.20 [2.25] patients/month; –12.09% change; *P* = .42). No significant difference was observed according to stage, although an increase was noted in the number of cases of stage II biliary tract cancer.

## Discussion

To our knowledge, this is the first study to evaluate whether the COVID-19 pandemic has affected the stage of gastrointestinal cancer at diagnosis in Japan. Additionally, this is the first study to evaluate the association of the COVID-19 pandemic with the cancer stage at diagnosis not only in the first phase of the pandemic (ie, March to June) but also until the end of 2020. Previous studies have shown that the numbers of patients decreased significantly for almost all cancer types,^10,11,15^ although we only observed significant decreases for gastric and colorectal cancers. Interestingly, during the state of emergency, the overall number of patients with newly diagnosed gastrointestinal cancer decreased, although subsequent increases resulted in nonsignificant changes during the study period for most cancer types. The number of endoscopes and return visits followed a similar course. However, the number of initial patient visits also increased after the state of emergency was declared but decreased significantly throughout the study period.

Stage-specific comparisons revealed significant decreases in the numbers of patients with stage I gastric cancer and stage 0 or I colorectal cancer. This may be related to a decrease in the number of screening-detected cases, given that there was a decrease in the number of gastrointestinal cancer cases that were detected by screening patients who were visiting the hospital for the treatment of other diseases. Specifically, this number was significantly decreased for gastric cancer cases. We postulate that the lack of significant difference in colorectal cancer may be due to the inclusion of patients with positive fecal occult blood tests as symptomatic in this cancer registry, even if the newly diagnosed cancer was early stage and not bleeding. Additionally, despite speculation that the number of cases identified by medical checkup would be decreased, we found almost no change. This may be because there were no restrictions on medical checkups in Japan, enabling patients to undergo medical checkups without interruption, despite the initial consultation having been postponed.

For colorectal cancer, there was a significant increase in the number of stage III cases. However, stage II colorectal cancer was significantly reduced, and stage II to IV colorectal cancer increased only slightly. Specifically, there is a possibility that stage II progressed to stage III because of the diagnosis delay related to the COVID-19 pandemic. Colorectal cancer is generally recognized as a slow-growing cancer, with prolonged times to doubling and progression, and years are typically needed for adenomas to become cancerous.^23,24^ However, the rate of progression is less clear for more advanced colorectal cancer. Several studies, including 2 systematic reviews,^25,26^ have evaluated the intervals from diagnosis to surgery and then to death. Among patients with colon cancer, overall survival decreases when the time from diagnosis to surgery exceeds 30 to 40 days, and overall survival among patients with rectal cancer decreases when the time from the end of neoadjuvant therapy to surgery exceeds 7 to 8 weeks.^25,26^ Other systematic reviews have also indicated that overall survival decreases when the time to surgery exceeds 12 weeks.^27^ Thus, 6 to 12 weeks may be a sufficient interval for colorectal cancer to progress, which would support our findings, as many Japanese patients would have had their colonoscopy delayed by more than 6 to 12 weeks because of the COVID-19 pandemic.

Gastric cancer has a poorer 5-year survival rate than colorectal cancer and is thought to progress more rapidly.^21^ The number of patients with stage IV gastric cancer increased by 11.36%, but there was no significant difference between the pre–COVID-19 period and the COVID-19 period, and unlike colorectal cancer, there was no increase in patients with stage III gastric cancer. This may be related to the fact that while the number of individuals with colorectal cancer is on the rise in Japan, the number of individuals with gastric cancer is on the decline due to the positive progress in the eradication of *Helicobacter pylori*.^21^ Additionally, gastric cancer is often found at stage IV instead of earlier because of the lack of subjective symptoms and a simple screening method, such as the fecal occult blood test. In this study, before COVID-19, patients with stage II gastric cancer made up only approximately one-third of those with stage II colorectal cancer. Therefore, it is highly possible that this study does not reflect the progression of gastric cancer.

Unlike gastric and colorectal cancers, pancreatic cancer is generally not detected via screening, and most patients present with symptoms, including abdominal pain, jaundice, weight loss, and new-onset diabetes.^28,29^ These factors may explain the lack of a significant change in the number of pancreatic cancer cases during the COVID-19 period. Pancreatic cancer is an aggressive malignant neoplasm with a reported tumor doubling time of 159 days and very poor overall survival.^30^ Therefore, we suspected that the greatest change would be observed for pancreatic cancer, although we failed to detect a significant difference. However, because we observed a decrease for localized disease and increases for regional and distant disease, significant differences might emerge over a prolonged observation period with a larger number of patients.

Because esophageal cancer can be detected via screening, we suspected that the number of patients with new diagnoses would decrease. However, we only observed a minimal change in the number of esophageal cancer cases. These results do not appear to reflect the effects of reduced screening, as national statistics indicate that esophageal cancer is only detected in 19.2% of patients with gastric cancer and in 16.3% of patients with colorectal cancer.^21^ Given that the esophagus does not have a serous membrane, esophageal cancer progresses more rapidly than gastric or colorectal cancer, which suggests that significant changes might be observed over a longer observation period with a larger number of patients (similar to the trend for pancreatic cancer).

No significant differences were observed overall or according to stage among patients with hepatocellular carcinoma. This may be related to the relative ease of identifying patients with a high risk of hepatocellular carcinoma and the large number of patients who are under surveillance. However, given that the greatest decrease was observed in the number of stage I hepatocellular carcinoma cases, it is possible that we did not identify enough patients with early-stage cancer. In this setting, surveillance intervals shorter than 6 months have limited utility and intervals of 12 months or longer may be too long to detect early-stage cancer.^31,32,33^ Therefore, the number of hepatocellular carcinoma cases may increase if patients who voluntarily dropped out of the surveillance program because of the COVID-19 pandemic undergo subsequent examinations at intervals of 12 months or longer.

Biliary tract cancer is typically detected based on easily noticed signs and symptoms, such as jaundice and abdominal pain.^34,35^ This may explain the lack of a significant change overall or according to stage among patients with biliary tract cancer. However, we only identified an increase in the number of stage II cases, which may suggest that the average tumor size had increased slightly.

### Limitations

Our study has several limitations. First, we only collected data until the end of 2020, and this duration may not be sufficient to fully clarify the effects of the COVID-19 pandemic, given the speed of cancer progression. Thus, more accurate results may be observed over a prolonged observation period. Second, we only collected data from 2 Japanese hospitals, which might limit the power of the analyses, and we did not consider interhospital differences. Thus, including a larger sample of patients from more institutions might provide more representative results. Third, since this retrospective study only counted the number of patients diagnosed per month, factors unrelated to the COVID-19 pandemic cannot be considered. However, in this specific medical area, there were no disasters, significant changes in the number of residents, new hospitals established, or significant changes in the number of staff. Still, to eliminate factors other than COVID-19 as much as possible, it is necessary to obtain risk data for the overall population of the survey area. Fourth, we only evaluated patients in Japan, and we cannot comment on whether differences might emerge in different regions. Particularly, it is possible that the COVID-19 pandemic had greater effects on cancer detection in regions with more widespread infection and/or without a universal health care system, such as the United States and Europe. It would also be useful for future studies to consider whether the COVID-19 pandemic is associated with changes in overall survival among patients with gastrointestinal cancers.

## Conclusions

During the COVID-19 pandemic in Japan, we observed that the number of patients diagnosed with stage I gastric and colorectal cancers decreased significantly. Additionally, a significant increase was observed in the number of patients diagnosed with stage III colorectal cancer. Given the ongoing nature of the COVID-19 pandemic, it may have further negative effects on patients with cancers that are asymptomatic and/or typically detected via screening. Thus, it is important to ensure that patients undergo necessary screening and surveillance without waiting for the pandemic to end, especially patients who require colonoscopy to potentially detect colorectal cancer. Moreover, given the speed of cancer progression, it is possible that the negative effects of the COVID-19 pandemic may become more pronounced over time, and further research is needed to better evaluate this issue.
